# End-of-life care in Germany between 2016 and 2020 – A repeated cross-sectional analysis of statutory health insurance data

**DOI:** 10.1186/s12904-024-01387-6

**Published:** 2024-04-20

**Authors:** Katharina van Baal, Melissa Hemmerling, Jona Theodor Stahmeyer, Stephanie Stiel, Kambiz Afshar

**Affiliations:** 1 Institute for General Practice and Palliative Care, Hannover Medical School, Carl-Neuberg-Straße 1, 30625 Hannover, Germany; 2 AOK Lower Saxony, Department for Health Services Research, Hildesheimer Str. 273, 30519 Hannover, Germany

**Keywords:** Palliative care, End-of-life care, Health services research, Outpatient care, Statutory health insurance data

## Abstract

**Background:**

The Hospice and Palliative Care Act of 2015 aimed at developing and regulating the provision of palliative care (PC) services in Germany. As a result of the legal changes, people with incurable diseases should be enabled to experience their final stage of life including death according to their own wishes. However, it remains unknown whether the act has impacted end-of-life care (EoLC) in Germany.

**Objective:**

The present study examined trends in EoLC indicators for patients who died between 2016 and 2020, in the context of Lower Saxony, Germany.

**Methods:**

Repeated cross-sectional analysis was conducted on data from the statutory health insurance fund AOK Lower Saxony (AOK-LS), referring to the years 2016–2020. EoLC indicators were: (1) the number of patients receiving any form of outpatient PC, (2) the number of patients receiving generalist outpatient PC and (3) specialist outpatient PC in the last year of life, (4) the onset of generalist outpatient PC and (5) the onset of specialist outpatient PC before death, (6) the number of hospitalisations in the 6 months prior to death and (7) the number of days spent in hospital in the 6 months prior to death. Data for each year were analysed descriptively and a comparison between 2016 and 2020 was carried out using t-tests and chi-square tests.

**Results:**

Data from 160,927 deceased AOK-LS members were analysed. The number of patients receiving outpatient PC remained almost consistent over time (2016 vs. 2020 *p* = .077). The number of patients receiving generalist outpatient PC decreased from 28.4% (2016) to 24.5% (2020; *p* < .001), whereas the number of patients receiving specialist outpatient PC increased from 8.5% (2016) to 11.2% (2020; *p* < .001). The onset of generalist outpatient PC moved from 106 (2016) to 93 days (2020; *p* < .001) before death, on average. The onset of specialist outpatient PC showed the reverse pattern (2016: 55 days before death; 2020: 59 days before death; *p* = .041).

**Conclusion:**

Despite growing needs for PC at the end of life, the number of patients receiving outpatient PC did not increase between 2016 and 2020. Furthermore, specialist outpatient PC is being increasingly prescribed over generalist outpatient PC. Although the early initiation of outpatient PC has been proven valuable for the majority of people at the end of life, generalist outpatient PC was not initiated earlier in the disease trajectory over the study period, as was found to be true for specialist outpatient PC. Future studies should seek to determine how existing PC needs can be optimally met within the outpatient sector and identify factors that can support the earlier initiation of especially generalist outpatient PC.

**Trial registration:**

The study “Optimal Care at the End of Life” was registered in the German Clinical Trials Register (DRKS00015108; 22 January 2019).

## Background

It is estimated that up to 90% of individuals at the end of life will require palliative care (PC) [1–5]. Due to the ageing society in Germany, the number of patients requiring PC is anticipated to continuously increase over the coming decades, bringing considerable challenges to the health care system and providers [6]. In light of this trend, PC offers have recently expanded, an increasing number of hospices and PC units have been established and financial compensation for PC services has improved.

In Germany, outpatient PC services include both generalist and specialist PC. Generalist outpatient PC has been offered since 2013. It is mainly provided by general practitioners (GPs) for patients with overall low symptom burden, and it is ideally initiated early in a patient’s disease trajectory [7, 8]. Specialist outpatient PC, on the other hand, has been offered since 2007, and can be initiated by both outpatient and inpatient physicians. Specialist outpatient PC is provided by an interdisciplinary team consisting of PC specialists (mainly doctors and nurses, if necessary social workers, psycho-oncologists, physical therapists and others), and it is usually offered to patients with complex symptoms and needs [9].

The German Hospice and Palliative Care Act of 2015 aimed at developing and regulating the provision of PC in Germany [10, 11]. The act explicitly introduced PC as part of standard care within the frameworks of statutory health insurance [12]. Among its targets for improvement, it focused on financing for hospice services, the expansion of generalist outpatient PC, networking between different service providers and contract closing for specialist outpatient PC [11]. During the implementation of the act, an agreement was reached to establish an intermediate level of outpatient PC [13] between generalist and specialist outpatient PC, but more aligned with the former. This form of outpatient PC is not well-established yet [14], but aims to close the existing gap between generalist and specialist outpatient PC and therefore improve (outpatient) care and allow patients to die in the environment of their choice.

Previous research has revealed that the number of patients receiving specialist outpatient PC at the end of life is increasing but highly variable between the federal states in Germany; however, no similar pattern has been noted for generalist outpatient PC [15–18]. Unfortunately, there are no data on trends in end-of-life care (EoLC) since the implementation of the German Hospice and Palliative Care Act until 2020, especially with regard to outpatient PC. Thus, the present study aimed at evaluating developments in EoLC for patients who died between 2016 and 2020, on the basis of selected EoLC indicators, drawing on statutory health insurance data for deceased individuals in Lower Saxony, Germany. The following research questions were addressed:

1. To what extent did the proportion of patients receiving outpatient PC in the last year of life change between 2016 and 2020?
2. How did the initiation of outpatient PC prior to death differ between 2016 and 2020?
3. How did the number and duration of hospitalisations in the 6 months prior to death differ between 2016 and 2020?

Three hypotheses were proposed for the comparison between 2016 and 2020:

1. The proportion of patients receiving outpatient PC in the last year of life would remain constant;
2. All forms of outpatient PC would be initiated at increasingly earlier stages in the disease trajectory (i.e. at a greater distance from death); and.
3. Hospitalisations in the 6 months prior to death would become less frequent and progressively shorter.

## Methods

### Study design

A repeated cross-sectional analysis of secondary data (i.e. statutory health insurance data) was performed. The description followed the RECORD statement (Reporting of studies Conducted using Observational Routinely-collected Data) [19] and the Memorandum Health Services Research in the last year of life [20]. The study was developed in the context of the research project “Optimal Care at the End of Life” (OPAL) [21], funded by the Innovation Fund of the Federal Joint Committee.

### Study population

The study data referred to insured members of the statutory health insurance fund AOK Lower Saxony (AOK-LS). AOK-LS is the largest health insurance provider in Lower Saxony, insuring more than 2.9 million people [22]. AOK-LS holds reliable data on approximately 36% of Lower Saxony residents, pertaining to sociodemographic information, outpatient and inpatient diagnoses, treatments and billing codes. As a federal state with both urban and rural demographics and infrastructure, Lower Saxony is comparable to other federal states in Germany and to Germany as a whole [23, 24].

The present analysis referred to data for AOK-LS members who died between 2016 and 2020. All members who were residents of Lower Saxony, aged at least 18 years at the time of death and continually insured in their year of death and the preceding calendar year were included in the analysis. Additionally, a valid diagnosis for at least one chronic progressive oncologic or non-oncologic disease in the last year of life was an inclusion criterion. Diagnoses of interest were predefined according to the *International Statistical Classification of Diseases and Related Health Problems – 10th Revision* (ICD-10) and the literature [1, 3]. Subsequently, an interdisciplinary expert panel comprised of physicians, nursing scientists, sociologists and health scientists revised the ICD-10 code list. Diagnoses from outpatient settings were considered valid if the associated ICD-10 codes were documented in at least two of the five quarters prior to death (including the quarter of death and four preceding quarters). For the inpatient sector, diagnoses were considered valid if at least one diagnosis (main or secondary) was coded in the last year of life [25]. This method including the ICD-10 code list has been described in more detail in previous studies [16, 26].

### Outcomes

The data were analysed with reference to EoLC indicators, as described in the literature [5, 16, 27]. In particular, the following EoLC indicators were considered:

1. Proportion of patients receiving any form of outpatient PC (i.e., generalist, specialist, intermediate) in the last year of life;
2. Proportion of patients receiving generalist outpatient PC in the last year of life;
3. Proportion of patients receiving specialist outpatient PC in the last year of life;
4. Onset of generalist outpatient PC before to death;
5. Onset of specialist outpatient PC before to death;
6. Number of hospitalisations in the 6 months prior to death; and.
7. Days spent in hospital in the 6 months prior to death.

Additional indicators of interest were the proportion of patients receiving an intermediate level of PC in the last year of life and the number of patients who died in hospital. The indicator regarding the proportion of patients receiving an intermediate level of PC in the last year of life referred to the period of 2017–2020, as this form of PC was only implemented in the fourth quarter of 2017 [13]. Accordingly, the results show a starting point and development of this form of PC in the first few years after its implementation. While patients with oncological diseases are offered special oncological PC (delivered by oncologists), this form of care was not considered in the analyses.

### Data analysis

Descriptive statistics for each year (i.e. frequency, mean, median) were calculated using the IBM Statistical Package for Social Sciences (SPSS) software, version 27. A statistical comparison between 2016 and 2020 was conducted using t-tests or chi-square tests, depending on the type of variable. P-values less than 0.05 were considered as statistically significant.

## Results

### Description of the study sample

**Fig. 1:**
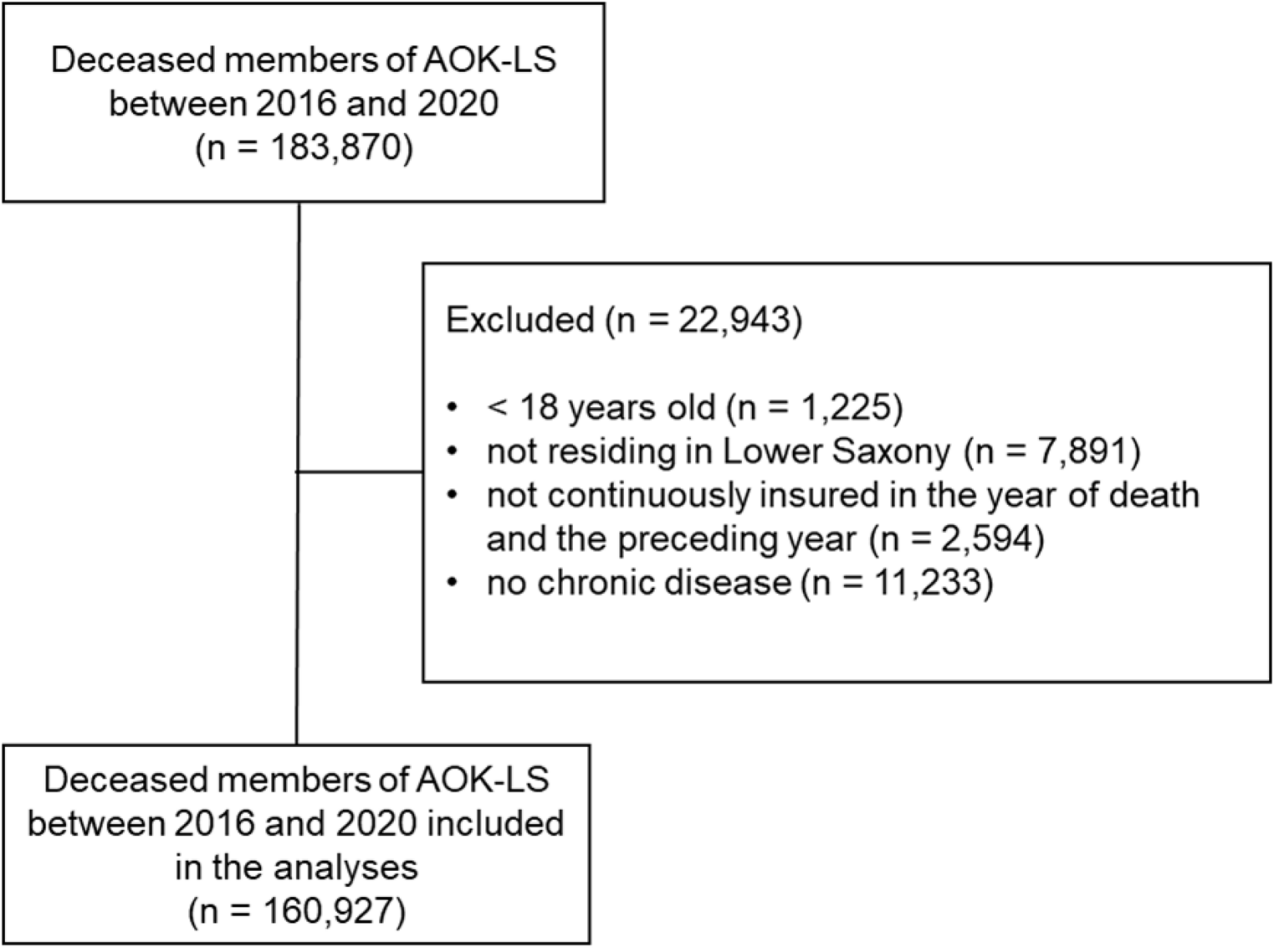
Flow chart of the deceased AOK-LS members

Data referring to 160,927 AOK-LS members who died between 2016 and 2020 were analysed (2016: 32,442; 2017: 31,833; 2018: 32,098; 2019: 31,394; 2020: 33,160; Fig. 1). Table 1 reports the demographic characteristics. In every year, the proportion of women was higher than that of men. Comparing 2016 to 2020, the sex distribution differed significantly (*p* < .001). The mean age at death ranged from 79.8 years in 2016 to 80.2 years in 2020 (*p* < .001). The most frequent disease groups were heart diseases, dementia/Alzheimer’s/senility/frailty diseases and respiratory diseases. Over the study period, heart diseases declined from 75.7% in 2016 to 74.5% in 2020 (*p* < .001); dementia/Alzheimer’s/senility/frailty diseases increased from 53.6% in 2016 to 54.5% in 2020 (*p* = .017) and respiratory diseases increased from 47.1% in 2016 to 49.9% in 2020 (*p* < .001).

**Table 1:** Demographic characteristics, *N* = 160,927 (2016: 32,442; 2017: 31,833; 2018: 32,098; 2019: 31,394; 2020: 33,160)

Characteristic		2016 n (%)	2017 n (%)	2018 n (%)	2019 n (%)	2020 n (%)	2016 to 2020 (p)**
Sex	Female	17,662 (54.4)	17,216 (54.1)	17,372 (54.1)	16,870 (53.7)	17,564 (53.0)	< 0.001
	Male	14,780 (45.6)	14,617 (45.9)	14,726 (45.9)	14,524 (46.3)	15,596 (47.0)	
Age group	18–50	781 (2.4)	582 (1.8)	588 (1.8)	571 (1.8)	628 (1.9)	< 0.001
	51–60	2,006 (6.2)	1,755 (5.5)	1,784 (5.6)	1,750 (5.6)	1,817 (5.5)	
	61–70	3,768 (11.6)	3,466 (10.9)	3,614 (11.3)	3,597 (11.5)	3,956 (11.9)	
	71–80	8,467 (26.1)	7,153 (22.5)	6,965 (21.7)	6,473 (20.6)	6,567 (19.8)	
	81–90	12,364 (38.1)	12,653 (39.7)	12,602 (39.3)	12,682 (40.4)	13,431 (40.5)	
	> 90	5,056 (15.6)	6,224 (19.6)	6,545 (20.4)	6,321 (20.1)	6,761 (20.4)	
Disease group*	HIV/AIDS	30 (0.1)	31 (0.1)	21 (0.1)	31 (0.1)	32 (0.1)	0.967
	Malignant neoplasms	11,809 (36.4)	10,758 (33.8)	10,793 (33.6)	10,771 (34.3)	12,327 (37.2)	0.041
	Heart diseases	24,546 (75.7)	23,463 (73.7)	23,387 (72.9)	23,018 (73.3)	24,704 (74.5)	< 0.001
	Cerebrovascular diseases	10,216 (31.5)	8,798 (27.6)	8,843 (27.6)	8,328 (26.5)	9,517 (28.7)	< 0.001
	Renal diseases	13,219 (40.7)	12,665 (39.8)	12,766 (39.8)	12,714 (40.5)	14,267 (43.0)	< 0.001
	Liver diseases	5,621 (17.3)	4,749 (14.9)	4,699 (14.6)	4,939 (15.7)	6,175 (18.6)	< 0.001
	Respiratory diseases	15,284 (47.1)	14,854 (46.7)	14,974 (46.7)	14,876 (47.4)	16,543 (49.9)	< 0.001
	Neurodegenerative diseases	2,279 (7.0)	1,937 (6.1)	1,963 (6.1)	1,853 (5.9)	2,159 (6.5)	0.009
	Dementia, Alzheimer’s, senility/frailty	17,382 (53.6)	14,825 (46.6)	15,054 (46.9)	14,539 (46.3)	18,076 (54.5)	0.017

*At least one valid diagnosis in this group

**Chi-squared test with a significance level of *p* ≤ .05

### Number of patients receiving outpatient PC

**Fig. 2:**
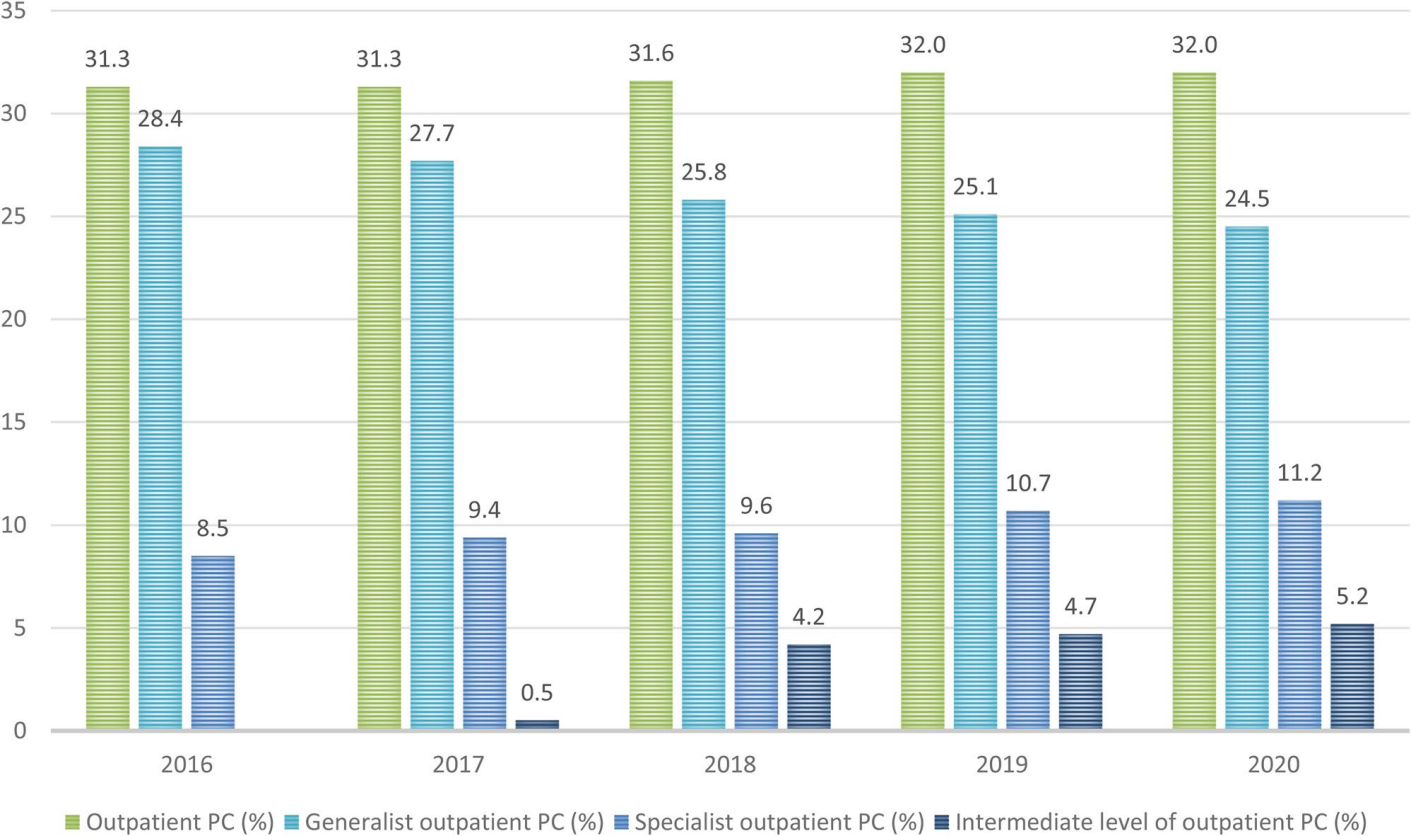
Proportion of the deceased AOK-LS members receiving any form of outpatient PC, generalist, specialist or an intermediate level of outpatient PC between 2016 and 2020

The proportion of patients receiving any type of outpatient PC (i.e. generalist, specialist, intermediate) remained almost constant between 2016 and 2020 (2016: 31.3%, 2017: 31.3%, 2018: 31.6%, 2019: 32.0%, 2020: 32.0%; 2016 vs. 2020 *p* = .077; Fig. 2). Over the study period, the number of patients receiving generalist outpatient PC decreased from 28.4% in 2016 to 24.5% in 2020 (*p* < .001). In contrast, the number of patients receiving specialist outpatient PC increased from 8.5% in 2016 to 11.2% in 2020 (*p* < .001; Fig. 2).

### Onset of outpatient PC

Relative to 2020, in 2016, generalist outpatient PC was initiated closer to death (2016 mean: 106 days, median: 48 days; 2020 mean: 93 days, median: 37 days; *p* < .001). Additionally, the proportion of patients whose generalist outpatient PC was initiated at least 8 months before death decreased over the study period (2016: 19.6%; 2020: 16.4%; Table 2), and the proportion of patients whose generalist outpatient PC was initiated 0–3 days before death increased from 11.6% in 2016 to 13.0% in 2020. In contrast, the onset of prescriptions for specialist outpatient PC was further from death in 2016 (mean: 55 days before death, median: 25 days) than in 2020 (mean: 59 days before death, median: 24) (*p* = .041). Simultaneously, the proportion of patients whose specialist outpatient PC was initiated at least 8 months before death rose (2016: 4.4%; 2020: 6.3%), but the proportion of patients whose specialist outpatient PC was initiated 0–3 days remained nearly consistent (2016: 14.4%; 2020: 14.9%).

### Hospitalisation

The proportion of patients with no hospital stay in the 6 months prior to death increased from 23.5% in 2016 to 26.1% in 2020 (*p* < .001; Table 2). Additionally, the mean number of hospital stays in the 6 months prior to death decreased from 1.6 (2016) to 1.5 (2020) (*p* < .001). Simultaneously, the number of treatment days spent in hospital decreased from 16.4 days in 2016 to 14.6 in 2020 (*p* < .001). Comparing 2016 to 2020, the proportion of patients who died in hospital decreased from 46.3 to 43.4% (*p* < .001). Prior to 2019, 46.3–47.7% of patients died in hospital. In 2020, this value decreased to 43.4%.

**Table 2:** EoLC indicators, *N* = 160,927 (2016: 32,442; 2017: 31,833; 2018: 32,098; 2019: 31,394; 2020: 33,160)

Indicator		2016 n (%)	2017 n (%)	2018 n (%)	2019 n (%)	2020 n (%)	2016 to 2020 (p)***
Generalist outpatient PC	Yes	9,207 (28.4)	8,814 (27.7)	8,270 (25.8)	7,880 (25.1)	8,109 (24.5)	< 0.001
	No	23,235 (71.6)	23,019 (72.3)	23,828 (74.2)	23,514 (74.9)	25,051 (75.5)	
Initiation of generalist outpatient PC before death (days) Subgroups 2016: *n* = 9,207 2017: *n* = 8,814 2018: *n* = 8,270 2019: *n* = 7,880 2020: *n* = 8,109	0–3	1,072 (11.6)	990 (11.2)	965 (11.7)	990 (12.6)	1,057 (13.0)	< 0.001
	4–10	1,111 (12.1)	1,090 (12.4)	1,048 (12.7)	1,036 (13.1)	1,178 (14.5)	
	11–20	926 (10.1)	943 (10.7)	851 (10.3)	795 (10.1)	910 (11.2)	
	21–30	669 (7.3)	644 (7.3)	572 (6.9)	597 (7.6)	574 (7.1)	
	31–60	1,244 (13.5)	1,270 (14.4)	1,134 (13.7)	1,073 (13.6)	1,138 (14.0)	
	61–120	1,207 (13.1)	1,146 (13.0)	1,064 (12.9)	1,035 (13.1)	977 (12.0)	
	121–240	1,175 (12.8)	1,081 (12.3)	1,057 (12.8)	959 (12.2)	948 (11.7)	
	≥ 241	1,803 (19.6)	1,650 (18.7)	1,579 (19.1)	1,395 (17.7)	1,327 (16.4)	
Specialist outpatient PC	Yes	2,771 (8.5)	2,982 (9.4)	3,097 (9.6)	3,349 (10.7)	3,724 (11.2)	< 0.001
	No	29,671 (91.5)	28,851 (90.6)	29,001 (90.4)	28,045 (89.3)	29,436 (88.8)	
Initiation of specialist outpatient PC before death (days) Subgroups 2016: *n* = 2,771 2017: *n* = 2,982 2018: *n* = 3,097 2019: *n* = 3,349 2020: *n* = 3,724	0–3	400 (14.4)	439 (14.7)	413 (13.3)	486 (14.5)	556 (14.9)	0.053
	4–10	475 (17.1)	538 (18.0)	504 (16.3)	575 (17.2)	628 (16.9)	
	11–20	391 (14.1)	428 (14.4)	441 (14.2)	440 (13.1)	541 (14.5)	
	21–30	265 (9.6)	286 (9.6)	311 (10.0)	326 (9.7)	314 (8.4)	
	31–60	446 (16.1)	489 (16.4)	543 (17.5)	533 (15.9)	577 (15.5)	
	61–120	405 (14.6)	413 (13.8)	405 (13.1)	484 (14.5)	512 (13.7)	
	121–240	266 (9.6)	262 (8.8)	310 (10.0)	307 (9.2)	362 (9.7)	
	≥ 241	123 (4.4)	127 (4.3)	170 (5.5)	198 (5.9)	234 (6.3)	
Intermediate level of outpatient PC**	Yes	-	158 (0.5)	1,350 (4.2)	1,491 (4.7)	1,715 (5.2)	-
	No	-	31,675 (99.5)	30,748 (95.8)	29,903 (95.3)	31,445 (94.8)	
Number of hospitalisations	0	7,611 (23.5)	6,811 (21.4)	7,042 (21.9)	6,887 (21.9)	8655 (26.1)	< 0.001
	1	10,784 (33.2)	10,815 (34.0)	10,837 (33.8)	10,673 (34.0)	11.109 (33.5)	
	2–3	10,657 (32.8)	10,693 (33.6)	10,755 (33.5)	10,409 (33.2)	10,327 (31.1)	
	≥ 4	3,390 (10.4)	3,514 (11.0)	3,464 (10.8)	3,425 (10.9)	3,069 (9.3)	
Number of treatment days in hospital Subgroups 2016: *n* = 24,831 2017: *n* = 25,022 2018: *n* = 25,056 2019: *n* = 24,507 2020: *n* = 24,505	1–3	3,067 (12.4)	3,007 (12.0)	3,113 (12.4)	3,051 (12.4)	3,276 (13.4)	< 0.001
	4–7	3,551 (14.3)	3,765 (15.0)	3,745 (14.9)	3,768 (15.4)	3,969 (16.2)	
	8–14	5,578 (22.5)	5,590 (22.3)	5,589 (22.3)	5,447 (22.2)	5,711 (23.3)	
	15–30	7,023 (28.3)	7,124 (28.5)	7,106 (28.4)	6,878 (28.1)	6,747 (27.5)	
	31–60	4,255 (17.1)	4,210 (16.8)	4,107 (16.4)	4,063 (16.6)	3,638 (14.8)	
	61–100	1,068 (4.3)	1,058 (4.2)	1,097 (4.4)	1,007 (4.1)	908 (3.7)	
	≥ 101	289 (1.2)	268 (1.1)	299 (1.2)	293 (1.2)	256 (1.0)	
Death in hospital	Yes	15,021 (46.3)	15,184 (47.7)	15.182 (47.3)	14,692 (46.8)	14,407 (43.4)	< 0.001
	No	17,421 (53.7)	16,649 (52.3)	16,916 (52.7)	16,702 (53.2)	18.753 (56.6)	

PC = palliative care; ^*^minor differences in totals due to rounding; ** the intermediate level of PC was implemented in 2017; ***Chi-squared test with a significance level of *p* ≤ .05

## Discussion

The present study conducted repeated cross-sectional analysis of EoLC indicators using statutory health insurance data from Lower Saxony, Germany for patients who died between 2016 and 2020, especially regarding outpatient PC. Trends in outpatient PC over a study period of 5 years were observed.

### Main results

The results showed that the proportion of individuals receiving outpatient PC prior to death remained relatively constant between 2016 and 2020. However, the number of patients receiving generalist outpatient PC decreased, while the number of patients receiving specialist outpatient PC increased over the study period. The average number of days before death at which generalist outpatient PC was initiated decreased between 2016 and 2020, whereas the opposite pattern was observed for specialist outpatient PC. The number of hospitalisations and the number of treatment days in hospital in the 6 months prior to death declined slightly over the study period. Altogether, despite growing needs for PC at the end of life, the number of patients receiving outpatient PC did not increase between 2016 and 2020, indicating that the recent legal changes to strengthen outpatient PC (e.g. the introduction of the German Hospice and Palliative Care Act in 2015) may be insufficient.

### Proportion of patients receiving outpatient PC

Between 2016 and 2020, approximately 30% of the study sample received outpatient PC. In 2017, the intermediate level of PC was established to strengthen the provision of outpatient PC. However, fewer individuals received outpatient PC at the end of life than indicated by estimates of need [3, 4, 28]. In contrast, the provision of specialist outpatient PC met estimates of need (10–20%). The increasing proportion of individuals receiving specialist outpatient PC over the study period can be considered a first step in the desired direction [15, 29]. However, with regard to specialist PC in Germany, challenges exist concerning accessibility, cost-effectiveness, patient-relevant outcomes, and structural characteristics [18, 30, 31] The slight decrease in the provision of generalist PC and the concomitant increase in the provision of specialist PC may indicate a shift between the different forms of care. One possible explanation for this may be that specialist outpatient PC is better integrated into practice. On the other hand, there may be a lack of clear distinction between generalist outpatient PC and the newly implemented intermediate level of PC, resulting in the over prescription of specialist outpatient PC. Another explanation for the present results may be the overall late initiation of PC in patient care trajectories, suggesting that, by the time PC is initiated, symptoms and problems might be highly complex, resulting in an increased need for specialist outpatient PC. The shortage of medical personnel may also contribute to this trend, especially within primary care [32]. Overall, the present results suggest that the aims of the 2015 Hospice and Palliative Care Act [10], especially regarding the facilitation and expansion of generalist outpatient PC, have not yet been met. With respect to the newly implemented intermediate level of outpatient PC, the literature suggests rather incomplete implementation, highlighting barriers and limits to its feasibility in daily practice [14, 17, 33, 34].

A majority of the surveyed population received generalist outpatient PC, which is mainly provided by GPs. Since 2020, GPs have been heavily burdened by numerous factors, including COVID-19 [35–39]. Specialist outpatient PC teams have also been severely loaded [40], and resources for outpatient PC might be collectively exhausted. Moreover, the number of outpatient PC providers has remained fairly constant. Of note, a discrepancy between billing data and actual care might exist and there might be an overlap between geriatric care and generalist outpatient PC in the care for elderly patients [41]. Thus, it is possible that, in the surveyed sample, outpatient PC was provided but no or non-PC codes (e.g. geriatric numbers) were used for remuneration. Ditscheid et al. [42] quantified the risk of underestimation and concluded that in 43.5% of their sample in Lower Saxony geriatric numbers but no codes for generalist outpatient PC were billed. It can therefore be assumed that a larger number of patients actually received PC and that the unmet PC needs are smaller than assumed.

Taken together, the present results align with previous evidence showing an increased use of specialist outpatient PC and a decreased use of generalist outpatient PC [15, 16, 42]. When interpreting the results, it must be considered that previous studies have found large regional differences in the use of outpatient PC, both between individual counties in Lower Saxony [42, 43] and between federal states in Germany [15, 18]. A recent analysis of German-wide statutory health insurance data with deceased persons who died between 2016 and 2019 described the use of PC over time. This analysis focused on regional differences and showed a slightly increased use of PC for Germany as a whole, which was not seen for Lower Saxony [42].

### Onset of outpatient PC

The early initiation of outpatient PC is associated with several positive outcomes [44, 45]. Specifically, it can improve quality of life at the end of life and reduce the number of hospital admissions. However, GPs often struggle to estimate prognosis and identify PC needs at an early stage [46, 47], due to prognostic uncertainty (particularly in relation to multimorbid patients and patients with non-oncological chronic diseases [47, 48]). Many GPs also find it difficult to talk to patients about death, especially when they have not seen those patients regularly and over the long term. More specifically, GPs report anxiety about initiating EoLC discussions, raising the topic of EoLC at the wrong time and creating emotional distress for patients and their relatives [46, 47]. GPs may require assistance to deal with these challenges. To this end, the Supportive and Palliative Care Indicators Tool (SPICT-DE™), adjusted for use by German GPs [49, 50], may facilitate the identification of patients with palliative needs and the (early) initiation of patient-centred PC measures [51, 52].

### Other EoLC indicators

The number of hospitalisations and the number of treatment days in hospital slightly decreased over the study period, in alignment with previous research [16]. In 2020, fewer patients died in an inpatient setting, but this reduction may have been influenced by the COVID-19 pandemic [36]. While hospitalisations at the end of life may be indicated and beneficial for some patients, they are often experienced as inappropriate and burdensome by patients, relatives and health care providers [53–55]. Recent changes to the German health care system may have affected the number of hospitalisations and days spent in inpatient treatment [56]. Furthermore, the overall number of hospitalisations at the end of life might be high because physicians often struggle to determine the need for hospital admission [55]. Strategies to avoid hospital admission at the end of life include regularly marking the approach of death (facilitating a shift in professionals’ mindset), guiding and monitoring patients and their families in a holistic way and maintaining continuity of treatment and care [57]. Nevertheless, statutory health insurance data are unable to show whether hospital stays were planned or avoidable.

### Strengths and limitations

The use of statutory health insurance data allowed several years to be considered without the risk of selection bias. AOK-LS is the largest statutory health insurance fund in Lower Saxony, and a large number of deceased members were included in the present analysis, representing a major strength of the research. The present findings for Lower Saxony are transferable to the Federal Republic of Germany, as the analysed AOK-LS members were comparable to the general population, regarding sex and age. However, individuals with a lower sociodemographic status might be overrepresented in the study population [23]. A major limitation of the study is that the results were based on routinely collected data, recorded for billing purposes. It is possible that health care providers offered generalist outpatient PC without remunerating these services with the health insurance fund, or using different codes for the remuneration. Furthermore, the specialist outpatient PC data only contained the date of prescription, and actual treatment by a specialised PC team may have been delayed or cancelled. Additionally, data regarding specialist outpatient PC only included prescriptions from outpatient settings, while specialised PC services prescribed by hospital doctors were not included. According to Ditscheid et al. [42], approximately one-quarter of all prescriptions for specialist outpatient PC are initiated in an inpatient setting; thus, the data used in this study may have underestimated the number of patients receiving specialist outpatient PC. Finally, a longitudinal analysis in the strict sense was not possible, since the population of deceased members differed in each year. Therefore, a repeated cross-sectional analysis was deemed most appropriate for assessing changes in EoLC indicators over time.

## Conclusions

Despite the growing need for PC at the end of life, the number of patients receiving outpatient PC between 2016 and 2020 remained relatively consistent. Thus, recent legal changes to strengthen the provision of outpatient PC (e.g. the introduction of the German Hospice and Palliative Care Act in 2015) may be insufficient. The findings suggest that specialist outpatient PC is being increasingly prescribed over generalist PC. Furthermore, although the early initiation of outpatient PC is associated with several positive patient outcomes, especially generalist outpatient PC was not initiated earlier in the patient trajectory over the study period. Further research should explore if (and how) existing PC needs can be met, and which factors might support the earlier initiation of PC. To counteract any further decrease in the provision of generalist outpatient PC, actions must be taken to counteract staff shortages in primary care and to increase awareness of PC needs among GPs.

## Data Availability

Data from the statutory health insurance fund AOK-LS are not publicly available due to data privacy protection regulations. Data may be made available upon reasonable request from the corresponding authors.

## Abbreviations

AOK-LS: AOK Lower Saxony (local statutory health insurance fund)
EoLC: end-of-life care
GPs: general practitioners
ICD-10: *International Statistical Classification of Diseases and Related Health Problems – 10th Revision*
OPAL: Optimal Care at the End of Life
PC: palliative care
RECORD: REporting of studies Conducted using Observational Routinely collected Data
SPICT-DE™: Supportive and Palliative Care Indicators Tool
